# Molecular epidemiology and genetic evolution of PRRSV ORF5 in Sichuan, Southwest China

**DOI:** 10.3389/fmicb.2026.1753001

**Published:** 2026-01-28

**Authors:** Jifeng Yu, Runmin Kang, Yi Qing, Qingsong Xu, Jing Xie, Ye Cao, Yonggang Ye, Lu Xiao, Lei Xie, Xiaoxue Xiang, Long Zhou, Changqing Yu, Yong Huang

**Affiliations:** 1 College of Veterinary Medicine, Sichuan Agricultural University, Chengdu, Sichuan, China; 2 Animal Genetic Breeding and Reproduction Key Laboratory of Sichuan Province, Sichuan Animal Science Academy, Chengdu, Sichuan, China; 3 Chengdu Livestock and Poultry Genetic Resources Protection Center, Chengdu, Sichuan, China; 4 College of Animal and Veterinary Sciences, Southwest Minzu University, Chengdu, Sichuan, China; 5 Bamboo Diseases and Pest Control and Resources Development Key Laboratory of Sichuan Province, Leshan Normal University, Leshan, China

**Keywords:** genetic evolution analysis, JXA1-like strain, NADC30-like strain, ORF5, PRRSV, VR2332-like strain

## Abstract

**Background:**

Porcine reproductive and respiratory syndrome virus (PRRSV) is a major economic threat to the global swine industry. In China, PRRSV undergoes continuous evolution through frequent mutation and recombination, driving the emergence of diverse genotypes. This is of particular concern in Sichuan Province, a major swine-producing region in Southwest China, where recurrent PRRSV outbreaks have led to substantial economic losses.

**Objectives:**

This study was designed to investigate the epidemiological patterns and genetic evolution of PRRSV in Sichuan Province by analyzing the ORF5 gene. We collected samples from five major swine-producing cities (Chengdu, Mianyang, Meishan, Ya’an, and Yibin) throughout 2024. Our objectives were to determine the lineage distribution, assess sequence diversity, and identify key amino acid variations among the circulating strains. The findings aim to provide a molecular basis for informing and refining regional PRRSV control strategies.

**Methods:**

A total of 658 suspected PRRSV samples were collected from large-scale pig farms across the five cities. Clinical samples mainly included serum and tissue samples (such as lung and lymph node tissues) obtained from pigs showing PRRS-like symptoms during routine diagnostic procedures. The ORF5 gene was amplified by RT-PCR, sequenced, and analyzed for nucleotide and amino acid homology. Phylogenetic trees were constructed using MEGA 11 and EvolView to determine lineage classification. GP5 amino acid sequences were further examined to identify key mutations within signal peptides, transmembrane regions, and neutralizing epitopes.

**Results:**

Among the 658 samples, 185 (28.16%) were PRRSV-positive. Phylogenetic analysis of 53 representative ORF5 sequences revealed that all isolates belonged to PRRSV-2: 20 samples were NADC30-like (37.73%) strains, 1 samples were NADC34-like (1.89%) strains, 3 samples were QYYZ-like (5.66%) strains, 17 samples were VR2332-like (32.08%) strains, and 12 samples were JXA1-like (22.64%) strains. Multiple amino acid substitutions and deletions were detected in the GP5 protein, particularly in signal peptides, neutralizing epitopes, and transmembrane domains, indicating extensive genetic diversity.

**Conclusion:**

Our findings confirm that NADC30-like strains constitute the predominant lineage circulating in Sichuan Province, with co-circulation of JXA1-like and strains with potential recombination signatures inferred from phylogenetic clustering. The prevalence of frequent mutations within GP5 antigenic regions underscores the substantial genetic diversity of PRRSV. These results emphasize the critical need for sustained molecular surveillance and the development of updated vaccines to effectively manage PRRS outbreaks in the region.

## Introduction

Porcine reproductive and respiratory syndrome (PRRS), caused by the PRRS virus (PRRSV), remains one of the most economically significant viral diseases affecting the global swine industry (Aundrup et al., 2024; Kikuti et al., 2024). The disease is characterized by reproductive failure in sows and severe respiratory distress in piglets, leading to substantial production losses worldwide (Shin et al., 2024; Suh and Chae, 2025). PRRSV is a single-stranded, positive-sense RNA virus belonging to the *Arteriviridae* family, known for its high mutation rate and frequent recombination events, which collectively drive its rapid genetic evolution and antigenic diversity (Tang et al., 2025).

Two major genotypes are recognized globally, PRRSV-1 (European type) and PRRSV-2 (North American type). These genotypes share approximately 60% nucleotide identity but exhibit significant differences in antigenicity and pathogenicity (Li G. et al., 2024; Li Y. et al., 2024). Since its initial introduction into China in the 1990s, PRRSV-2 has become the dominant genotype circulating in Chinese swine herds. Over the past decades, multiple lineages have emerged and co-circulated, with frequent recombination events contributing to the complex genetic landscape of the virus (Zhang et al., 2024). Recent molecular epidemiological studies indicate that lineages 1 (including NADC30-like and NADC34-like), 3 (QYYZ-like), 5 (VR2332-like), and 8 (JXA1-like, often associated with highly pathogenic PRRSV, HP-PRRSV) are currently prevalent across China (Liu et al., 2025; Zhang et al., 2024). For instance, a large-scale survey in northern China involving 174 PRRSV-2 isolates revealed that ORF5 gene sequences clustered predominantly into sub-lineages L1.8, L1.5, and L8, with widespread amino acid polymorphisms observed in neutralizing and decoy epitopes (Yuan et al., 2025).

The ORF5 gene, which encodes the major envelope glycoprotein GP5, represents one of the most variable and immunologically critical regions of the PRRSV genome. GP5 harbors the principal neutralizing epitope, a decoy epitope, several N-linked glycosylation sites, and transmembrane domains, all of which play essential roles in viral infectivity, immune evasion, and vaccine-induced protection (Luo et al., 2023a; Li G. et al., 2024; Li Y. et al., 2024; Rowland and Brandariz-Nuñez, 2024). Amino acid substitutions at key positions, such as residues 13 and 151, have been closely associated with altered virulence and the ability of field strains to escape host immune responses (Jian et al., 2025; Kang et al., 2025). A previous study focusing on Chengdu area isolates (2022–2023) identified several substitutions in ORF5 that were suggestive of vaccine-derived recombination events (Zhong et al., 2025). It is acknowledged that whole-genome analysis would provide a more comprehensive characterization of PRRSV recombination events by capturing cross-genomic genetic exchanges; however, ORF5 was selected as the focus of this study due to its well-established utility in accurate lineage tracking and identification of key antigenic variations—information directly relevant to guiding regional PRRSV surveillance and control strategies.

As a major swine-producing region in southwestern China, Sichuan faces persistent challenges from diverse and evolving PRRSV strains. A recent epidemiological survey (2021–2023) reported a high PRRSV positivity rate of 39.74% in the province, highlighting a complex and dynamic lineage distribution along with considerable genetic variation (Huang et al., 2024a,b). However, data on the most recent evolutionary trends (2024) of PRRSV in Sichuan remain limited, particularly regarding ORF5 gene diversity and GP5 amino acid variation across multiple key pig-producing cities.

To address this gap, the present study was designed to conduct a comprehensive epidemiological and genetic analysis of PRRSV in five major pig-producing cities in Sichuan Province, including Chengdu, Mianyang, Meishan, Ya’an, and Yibin throughout 2024. By combining virus detection, ORF5 gene sequencing, phylogenetic reconstruction, and GP5 amino acid variation profiling, this study aims to clarify the current lineage composition, genetic diversity, and key mutation patterns of circulating PRRSV strains. These findings are expected to enhance molecular surveillance databases and provide a scientific basis for the development of targeted prevention and control strategies in Sichuan.

## Materials and methods

### Sample collection

From January to December 2024, an epidemiological surveillance program for PRRSV was implemented across five major swine-producing cities in Sichuan, including Chengdu, Mianyang, Meishan, Ya’an, and Yibin. A total of 658 clinical samples were collected from pigs showing typical PRRS-like symptoms on large-scale commercial farms in these regions. The collected clinical samples mainly included serum and tissue samples (such as lung and lymph node tissues) obtained during routine diagnostic procedures. The sampling strategy was designed to capture a representative profile of the circulating virus by including multiple pig production stages and tissue types, as detailed in Table 1.

**Table 1:** Distribution of suspected PRRSV samples from five cities in Sichuan.

Year	Region	Suspected cases	Age (years)
2024	Chengdu	168	0–1
2024	Mianyang	193	1–2
2024	Meishan	112	1–2
2024	Ya’an	76	1–2
2024	Yibin	109	0–2
2024	Total	658	1–2

### Primer design and synthesis

To amplify the PRRSV ORF, we designed specific primers using Primer Premier 5.0 after aligning reference sequences from GenBank. The primers were synthesized by Qingke Biotechnology Co., Ltd. (Chengdu, China), and their sequences are listed in Table 2.

**Table 2:** Primers targeting the PRRSV ORF5.

Name	Primer sequence (5′ → 3′)	Product size (bp)
ORF5	F: AGCCTGTCTTTTTGCCATTCT R: CTTTTGTGGAGCCGTGCTATC	628

### Preparation of PRRSV cDNA

Viral RNA was extracted from the collected samples using a magnetic bead-based Viral DNA/RNA Extraction Kit (Compurify, Changzhou, China) according to the manufacturer’s instructions. Subsequently, the extracted RNA was reverse-transcribed into complementary DNA (cDNA) using an All-In-One 5 × RT MasterMix kit (Abm, Vancouver, Canada).

### ORF5 amplification, cloning, and sequencing

The ORF5 gene was amplified by reverse transcription polymerase chain reaction (RT-PCR) using the primer pairs listed in Table 2. The thermal cycling protocol consisted of an initial denaturation at 95 °C for 3 min; 35 cycles of denaturation at 95 °C for 25 s, annealing at 55 °C for 25 s, and extension at 72 °C for 15 s; followed by a final extension at 72 °C for 5 min. The amplification products were verified by electrophoresis on a 2.0% agarose gel stained with ethidium bromide. Subsequently, one to two representative PCR products from each pig farm were purified, cloned into a pMD18-T vector (Takara, Japan), and transformed for propagation. The resulting recombinant plasmids were sequenced bidirectionally by Qingke Biotechnology Co., Ltd. (Chengdu, China).

### ORF5 homology and phylogenetic analysis

One representative ORF5-positive sample from each pig farm per city was selected for sequencing. Representative samples were selected based on clear and specific RT-PCR amplification results. When multiple ORF5-positive samples were obtained from the same pig farm, sequences exhibiting 100% nucleotide identity were considered redundant and excluded to avoid overrepresentation. Multiple sequence alignment was conducted using the Clustal W algorithm within MegAlign software (DNASTAR v7.1.0). To avoid redundancy, sequences exhibiting 100% identity from the same farm were excluded from subsequent analyses.

A final dataset was compiled, comprising representative ORF5 sequences from this study (Supplementary Table S2) and reference strains of major PRRSV lineages (sublineages 1.5, 1.8, 3, 5, 8, and PRRSV-1) retrieved from GenBank (Supplementary Table S1). Pairwise nucleotide identity between field isolates and reference strains was calculated using MegAlign.

A phylogenetic tree was reconstructed from the aligned ORF5 sequences using MEGA 11, employing the neighbor-joining method with 1,000 bootstrap replicates to assess node support. The resulting tree was visualized and annotated in EvolView 3.0, where distinct colors and shapes were used to represent phylogenetic lineages and geographical origins, respectively.

Sequence variation analysis was performed using MegAlign, and amino acid polymorphisms in the encoded GP5 protein were graphically represented.

### PRRSV GP5 amino acid analysis

Amino acid sequence analysis was performed on ORF5 sequences derived from the 53 PRRSV isolates obtained in this study, along with six reference sequences downloaded from GenBank (Supplementary Table S1). The nucleotide sequences were translated into their corresponding amino acid sequences using MEGA 11 software. Multiple sequence alignment was conducted to identify amino acid substitutions, insertions, and deletions among the studied isolates and reference strains.

To characterize genetic variability in the encoded GP5 protein, the aligned amino acid sequences were further analyzed and visualized using the MegAlign module of DNASTAR Lasergene software. Specific emphasis was placed on key functional regions, including the signal peptide, transmembrane domains, the primary neutralizing epitope (PNE), and known B-cell and T-cell epitopes. Amino acid variations within these domains were systematically identified and compared across different PRRSV lineages to delineate potential lineage-specific mutation patterns.

## Results

### Detection of PRRSV by RT-PCR in five cities of Sichuan

**Figure 1:**
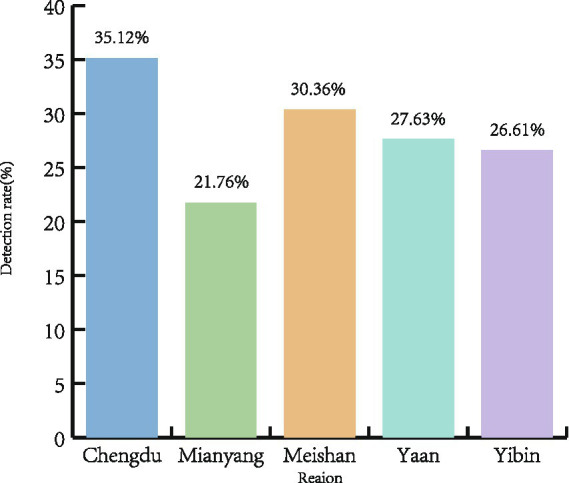
Geographic distribution of Porcine Reproductive and Respiratory Syndrome Virus (PRRSV) detection rates in five cities of Sichuan Province, China (January to December 2024). A total of 658 clinical samples were collected from large-scale pig farms in Chengdu, Mianyang, Meishan, Ya’an, and Yibin. PRRSV was detected by RT-PCR targeting the ORF5 gene. The bar chart illustrates regional variation in positivity rates, with Chengdu showing the highest rate (35.12%) and Mianyang the lowest (21.76%).

A total of 658 clinical samples were collected from large-scale pig farms across five major swine-producing cities in Sichuan Province between January 2024 and December 2024 and were subjected to RT-PCR detection targeting the PRRSV ORF5 gene. Among them, 185 samples tested positive, yielding an overall positivity rate of 28.16% (185/654). The detection rates, however, varied substantially among the surveyed regions (Table 3; Figure 1). The highest rate was observed in Chengdu (35.12%), followed by Meishan (30.36%), Ya’an (27.63%), and Yibin (26.61%), whereas Mianyang showed the lowest prevalence at 21.76%.

**Table 3:** PRRSV prevalence in five cities of Sichuan (2024).

Region	Number of samples	Number of positive samples	Detection rate (%)
Chengdu	168	59	35.12
Mianyang	193	42	21.76
Meishan	112	34	30.36
Ya’an	76	21	27.63
Yibin	109	29	26.61
Total	658	185	28.16

### Phylogenetic and genetic identity analysis of the PRRSV ORF5

The nucleotide and deduced amino acid sequences of the ORF5 gene from 53 PRRSV isolates (Supplementary Table S2) were compared with seven reference strains (Supplementary Table S1) to assess sequence homology. Nucleotide sequence identities between the isolates and reference strains were as follows: 82.6–99.7% with JXA1-like strains, 83.4–94.9% with CH-1a-like, 82.3–99.8% with VR2332-like, 80.9–89.9% with QYYZ-like, 85.1–94.5% with NADC30-like, 84.6–94.9% with NADC34-like, and 61.2–63.3% with the PRRSV-1 LV strain. Among the 53 isolates, nucleotide identity ranged from 81.6 to 99.8%.

At the amino acid level, the GP5 protein showed identities of 80.6–98.5% with JXA1-like strains, 80.6–92.0% with CH-1a-like, 77.6–99.0% with VR2332-like, 79.1–92.0% with QYYZ-like, 83.1–94.0% with NADC30-like, 83.6–93.0% with NADC34-like, and 52.2–57.2% with the PRRSV-1 LV strain. Inter-isolate amino acid identity varied between 78.1 and 99.5%.

**Figure 2:**
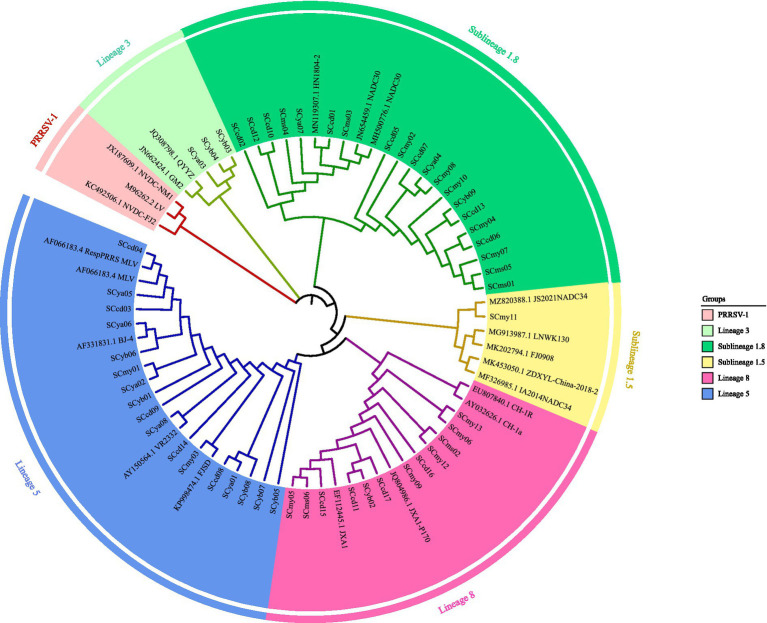
Phylogenetic analysis of PRRSV based on ORF5 gene sequences. The tree was constructed by the neighbor-joining method in MEGA 11 using sequences from 53 field isolates (Supplementary Table S2) and 22 reference strains (Supplementary Table S1), with 1,000 bootstrap replicates. Branches are colored by PRRSV lineage (L1.5, L1.8, L3, L5, L8, and PRRSV-1), and shapes denote the source of each sequence.

To elucidate the evolutionary relationships among the isolates, a phylogenetic tree was constructed using the neighbor-joining method in MEGA 11 with 1,000 bootstrap replicates, based on ORF5 sequences from the 53 isolates (Supplementary Table S2) and 22 reference strains from the NCBI database (Supplementary Table S1). All isolates clustered within the PRRSV-2 genotype and were classified into the following lineages: 20 isolates (37.73%) in lineage 1.8 (NADC30-like), one (1.89%) in lineage 1.5 (NADC34-like), three (5.66%) in lineage 3, 17 (32.08%) in lineage 5, and 12 (22.64%) in lineage 8 (Figure 2). Notably, the co-circulation of these diverse lineages creates opportunities for genetic recombination, although formal recombination analysis (e.g., using RDP4 software) was not performed in this study.

### Amino acid sequence analysis of PRRSV GP5

**Figure 3:**
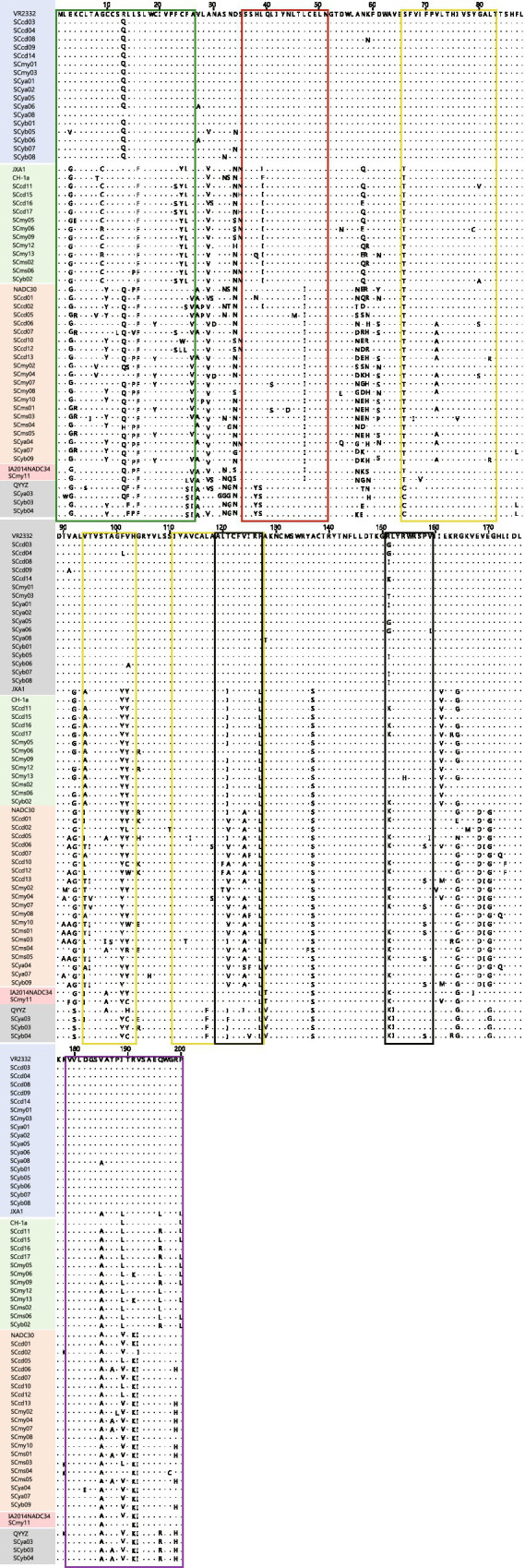
Distribution of amino acid mutations in the GP5 protein of PRRSV field isolates. Graphical representation of residue variations across key structural and immunogenic domains of GP5. Annotations are colored as follows: green, signal peptide; red, primary neutralizing epitope; yellow, transmembrane domain; black, T-cell epitope; purple, B-cell epitope.

The GP5 protein, encoded by the ORF5 gene, represents the most structurally complex and variable structural protein of PRRSV. Frequent amino acid deletions, insertions, and substitutions in GP5 have been widely reported to contribute to viral antigenic diversity and immune evasion (Luo et al., 2023b). In this study, analysis of GP5 sequences from 53 PRRSV isolates revealed no insertions; however, 12 isolates from lineage 1.8 exhibited deletions, 11 at position 32 and one at position 33 (Figure 3).

Positions 13 and 151 in GP5 are recognized as virulence-associated residues, with the presence of arginine (R) at both sites generally indicative of high pathogenicity (Allende et al., 2000; Yin et al., 2021). In the present isolates, an R^13^ → Q^13^ substitution was observed in 16 of 17 VR2332-like isolates, 1 of 3 QYYZ-like isolates, and 18 of 20 NADC30-like isolates. Mutations at position 151 were identified in 32 isolates, including R^151^ → K^151^ in 4 of 12 JXA1-like/CH-1a-like strains and 14 of 21 lineage 1 strains (NADC30-like and NADC34-like), while diverse substitutions (R^151^ → (G/I/K/T)^151^) were detected in 11 of 17 VR2332-like isolates. Only nine isolates—eight JXA1-like and one VR2332-like—retained R at both positions 13 and 151 (Figure 3).

Amino acid 137 (A^137^) serves as a molecular marker for distinguishing wild-type strains from vaccine strains such as VR2332, MLV, and RespPRRS/Repro (Wesley et al., 1998; Yin et al., 2021). With the exception of the 17 lineage 5 (VR2332-like) isolates, none of the field strains in this study exhibited the A^137^ residue characteristic of vaccine-derived strains. Furthermore, extensive amino acid variations were identified across multiple functional domains of GP5, including the signal peptide (aa 1–26), decoy epitope (aa 27–30), primary neutralizing epitope (aa 36–51), transmembrane region (aa 66–83), T-cell epitope (aa 119–127), and B-cell epitope region (aa 179–200).

In the signal peptide region, lineage 1.8 (NADC30-like) and lineage 1.5 (NADC34-like) isolates exhibited several substitutions: K^4^ → R^4^ in 6 of 21 isolates, Y^10^ → C^10^ in 7 of 21, and A^26^ → V^26^ in 16 of 21. All 12 JXA1-like/CH-1a-like isolates showed a G^9^ → (C/R)^9^ substitution. Within the decoy epitope, L^28^ → P^28^ was observed in 3 of 20 NADC30-like isolates, and N^30^ → D/S^30^ occurred in 5 of 53 isolates.

In the primary neutralizing epitope region, substitutions included H^38^ → (Q/Y/N)^38^ in 5 isolates, L^39^ → (I/S)^39^ in 16 isolates, L41S in 2 of 20 NADC30-like isolates, and L^45^ → M^45^ in one NADC30-like isolate. At position 47, leucine (L) was conserved in VR2332-like, JXA1-like/CH-1a-like, and QYYZ-like isolates, whereas isoleucine (I) was present in all NADC30-like and NADC34-like strains.

In the first transmembrane region (aa 66–83), V^72^ → A^72^ was identified in 12 of 21 lineage 1 isolates. Within the T-cell epitope (aa 119–127), I^121^ → V^121^ was observed in 16 of 21 lineage 1 isolates. In the B-cell epitope region (aa 179–200), R^199^ → H^199^ occurred in 9 isolates (including 3 QYYZ-like and 6 NADC30-like/NADC34-like strains), and P^200^ → L^200^ was detected in 9 of 12 JXA1-like/CH-1a-like isolates (Figure 3).

Notably, several lineage-specific mutation patterns were identified. The three QYYZ-like isolates analyzed in this study harbored previously reported substitutions, F^25^ → S^25^, A^26^ → I^26^, H^38^ → Y^38^, L^39^ → S^39^, T^66^ → C^66^, and L^152^ → I^152^, as well as a novel substitution, F^117^ → L^117^, which has not been documented in previous studies.

## Discussion

PRRS continues to impose severe economic losses on the global swine industry, largely due to the persistent evolution of its causative agent, PRRSV. As an RNA virus with high mutation and recombination rates, PRRSV maintains considerable genetic diversity, leading to the co-circulation of multiple lineages and recurrent outbreaks in China since its introduction in 1996 (Cui et al., 2024; Gong et al., 2024; Magalhães et al., 2025; Mei et al., 2025; Taira et al., 2025; Wu et al., 2024).

National surveillance data illustrate the complexity of PRRSV epidemiology. In northern China (2021–2023), a positivity rate of 18.42% was reported, with lineages 1.8 (NADC30-like), 1.5 (NADC34-like), and 8 (JXA1-like) predominating (Yuan et al., 2025). Notably, 24 of 27 sequenced isolates were recombinants, mostly involving lineage 1.8 as a backbone (Yuan et al., 2025). Similarly, novel recombinant strains derived from NADC30-like and JXA1-like viruses have been identified in Southwest China, displaying distinct pathogenic profiles (Huang et al., 2024a,b; Zhang et al., 2025). These findings establish lineage 1 viruses as key “recombination donors” that promote diversification through genetic exchange with vaccine and field strains (Wei et al., 2025).

In eastern China, a five-year study (2017–2022) reported 24% PRRSV positivity, with lineages 1, 3, 5, and 8 all detected (Zhou et al., 2022). GP5 and Nsp2 protein analyses revealed widespread amino acid variations across neutralizing and decoy epitopes, indicative of immune-driven evolution (Zhou et al., 2022). In Shandong, NADC30-like strains dominated, with emerging NADC34-like variants showing mutations in GP5 residues linked to virulence and immune evasion (Li et al., 2022). Together, these patterns support a model of “multi-source parallel adaptation,” in which lineage 1 strains exhibit high evolutionary flexibility under vaccination pressure.

In Sichuan Province, earlier surveillance (2019–2021) indicated a PRRSV positivity rate of 55.61%, dominated by JXA1-like (44.74%) and NADC30-like (17.54%) strains (Jiang et al., 2023). By 2021–2022, Chengdu reported a lower detection rate (11.42%) but continued circulation of sublineages 8.7, 5.1, 3.0, and 1.8, with mutations at residues 13 and 151 of GP5 (Zhong et al., 2025). Our 2024 data show a moderate positivity rate of 28.16%, suggesting a balance between biosecurity improvements and viral adaptation. Co-circulation of multiple lineages implies a heterogeneous viral ecology influenced by vaccination, partial immunity, and animal movement. Co-circulation of multiple distinct lineages (1.5, 1.8, 3, 5, and 8) in the same geographic region creates favorable conditions for potential recombination events, although direct evidence of recombination was not confirmed in this study due to the lack of whole-genome sequencing and formal recombination analysis.

The overall PRRSV positivity rate observed in this study (28.16%) was intermediate compared with previously reported rates from other regions and periods. Specifically, it was higher than the rate reported in Northern China (18.42%) and comparable to that reported in Eastern China (approximately 24%), but substantially lower than the positivity rate reported in earlier studies from Sichuan Province (55.61%). This pattern suggests that although PRRSV circulation in Sichuan remains active, the overall infection pressure in 2024 may have declined compared with earlier periods, possibly reflecting improvements in biosecurity measures and herd management, while continued viral evolution and lineage co-circulation sustain ongoing transmission.

Among 53 sequenced isolates, NADC30-like strains were most prevalent (37.73%), followed by VR2332-like (32.08%), JXA1-like (22.64%), and QYYZ-like (5.66%), consistent with the national shift toward lineage 1 dominance (Jian et al., 2025). GP5 protein analysis revealed deletions at positions 32–33 in 12 NADC30-like isolates and recurrent mutations at virulence-associated sites R^13^ and R^151^. These changes cluster in functional regions such as the signal peptide and neutralizing epitope (aa 36–52), suggesting positive selection. Similar mutation profiles reported across China imply convergent evolution aiding immune escape and possibly favoring a “low-virulence, high-transmissibility” phenotype (Fang et al., 2022; Huang et al., 2024a,b; Yuan et al., 2025).

Neutralizing epitope residues such as H^38^, L^39^, Q^40^, L^41^, and N^44^ also showed substitutions linked to altered antibody recognition and reduced vaccine efficacy (Huang et al., 2025; Dey et al., 2024; Zhou et al., 2023). The increasing complexity of mutations in NADC30-like and NADC34-like strains highlights their adaptive advantage in vaccinated herds (Wu et al., 2024; Zhang et al., 2025). Recombinants between lineages 1.8 and 8.7 have been documented in previous studies to broaden viral diversity, and the concurrent circulation of these lineages in our study suggests a similar potential for recombination events to occur (Zhang et al., 2024; Zhu et al., 2025).

In light of these findings, it is clear that current MLV vaccines often provide only partial protection against divergent field strains (Mebumroong et al., 2025; Yan et al., 2024). Enhancing genomic surveillance and developing lineage-tailored vaccines are thus critical to controlling PRRSV spread and minimizing its impact on swine production.

## Conclusion

In summary, this study demonstrates that PRRSV continues to circulate at high prevalence in Sichuan Province, with NADC30-like strains representing the dominant genotype. The co-circulation of multiple diverse lineages (1.5, 1.8, 3, 5, and 8) implies a potential for recombination, although formal recombination analysis was not conducted in this study. The frequent amino acid substitutions and deletions identified in the GP5 protein reflect ongoing viral evolution and adaptive changes under immune pressure, which are likely to undermine the efficacy of existing vaccines. Sustained molecular surveillance and the timely development of updated vaccines matched to circulating strains are therefore critical for effective PRRSV control and mitigating its impact on swine production.

## Data Availability

The authors selected the following statement: The datasets presented in this study can be found in online repositories. The names of the repository/repositories and accession number(s) can be found in the article/Supplementary material.
