# Supplementary material for: Molecular epidemiology and genetic evolution of PRRSV ORF5 in Sichuan, Southwest China
Source: Front Microbiol. 2026 Jan 28;17:1753001. doi: 10.3389/fmicb.2026.1753001 (PMC12891231; doi:10.3389/fmicb.2026.1753001)
Supplement: Supplementary file 1 [file Table_1.DOCX]

**Table S1** Reference sequences of PRRSV ORF5 gene

| **NO.** | **Name** | **Lineage** | **Accession number** | **NO.** | **Name** | **Lineage** | **Accession number** |
| --- | --- | --- | --- | --- | --- | --- | --- |
| 1 | QYYZ | 3 | JQ308798.1 | 12 | NADC30 | 1.8 | JN654459.1 |
| 2 | GM2 | 3 | JN662424.1 | 13 | NADC30 | 1.8 | MH500776.1 |
| 3 | JXA1 | 8 | EF112445.1 | 14 | HN1804-2 | 1.8 | MN119307.1 |
| 4 | JXA1-P170 | 8 | JQ804986.1 | 15 | IA2014NADC34 | 1.5 | MF326985.1 |
| 5 | CH-1a | 8 | AY032626.1 | 16 | LNWK130 | 1.5 | MG913987.1 |
| 6 | CH-1R | 8 | EU807840.1 | 17 | FJ0908 | 1.5 | MK202794.1 |
| 7 | VR2332 | 5 | AY150564.1 | 18 | JS2021NADC34 | 1.5 | MZ820388.1 |
| 8 | BJ-4 | 5 | AF331831.1 | 19 | ZDXYL-China-2018-2 | 1.5 | MK453050.1 |
| 9 | MLV | 5 | AF066183.4 | 20 | NVDC-FJ2 | / | KC492506.1 |
| 10 | FJSD | 5 | KP998474.1 | 21 | NVDC-NM1 | / | JX187609.1 |
| 11 | RespPRRS MLV | 5 | AF066183.4 | 22 | LV | / | M96262.2 |
