# Supplementary material for: Molecular epidemiology and genetic evolution of PRRSV ORF5 in Sichuan, Southwest China
Source: Front Microbiol. 2026 Jan 28;17:1753001. doi: 10.3389/fmicb.2026.1753001 (PMC12891231; doi:10.3389/fmicb.2026.1753001)
Supplement: Supplementary file 2 [file Table_2.DOCX]

**Table S2 Information on the clinical specimens and the amplifed sequences in the present study**

| **NO.** | **Designation** | **Accession no.** | **NO.** | **Designation** | **Accession no.** |
| --- | --- | --- | --- | --- | --- |
| 1 | SCcd01 | PX496758 | 28 | SCmy11 | PX496785 |
| 2 | SCcd02 | PX496759 | 29 | SCmy12 | PX496786 |
| 3 | SCcd03 | PX496760 | 30 | SCmy13 | PX496787 |
| 4 | SCcd04 | PX496761 | 31 | SCms01 | PX496788 |
| 5 | SCcd05 | PX496762 | 32 | SCms02 | PX496789 |
| 6 | SCcd06 | PX496763 | 33 | SCms03 | PX496790 |
| 7 | SCcd07 | PX496764 | 34 | SCms04 | PX496791 |
| 8 | SCcd08 | PX496765 | 35 | SCms05 | PX496792 |
| 9 | SCcd09 | PX496766 | 36 | SCms06 | PX496793 |
| 10 | SCcd10 | PX496767 | 37 | SCya01 | PX496794 |
| 11 | SCcd11 | PX496768 | 38 | SCya02 | PX496795 |
| 12 | SCcd12 | PX496769 | 39 | SCya03 | PX496796 |
| 13 | SCcd13 | PX496770 | 40 | SCya04 | PX496797 |
| 14 | SCcd14 | PX496771 | 41 | SCya05 | PX496798 |
| 15 | SCcd15 | PX496772 | 42 | SCya06 | PX496799 |
| 16 | SCcd16 | PX496773 | 43 | SCya07 | PX496800 |
| 17 | SCcd17 | PX496774 | 44 | SCya08 | PX496801 |
| 18 | SCmy01 | PX496775 | 45 | SCyb01 | PX496802 |
| 19 | SCmy02 | PX496776 | 46 | SCyb02 | PX496803 |
| 20 | SCmy03 | PX496777 | 47 | SCyb03 | PX496804 |
| 21 | SCmy04 | PX496778 | 48 | SCyb04 | PX496805 |
| 22 | SCmy05 | PX496779 | 49 | SCyb05 | PX496806 |
| 23 | SCmy06 | PX496780 | 50 | SCyb06 | PX496807 |
| 24 | SCmy07 | PX496781 | 51 | SCyb07 | PX496808 |
| 25 | SCmy08 | PX496782 | 52 | SCyb08 | PX496809 |
| 26 | SCmy09 | PX496783 | 53 | SCyb09 | PX496810 |
| 27 | SCmy10 | PX496784 |  |  |  |
